# Identification of *Klebsiella pneumoniae *that produces β-Lactamase bla_KPC _gene

**DOI:** 10.1186/1753-6561-8-S4-P26

**Published:** 2014-10-01

**Authors:** Julio Henrique Ferreira de Sá Queiroz, Kesia Esther Silva, Wirlaine Glauce Maciel, Maisa Estopa Correa, Flávia Patussi Correia Sacchi, Fábio Juliano Negrão, Júlio Henrique Rosa Croda, Peceu Magyve Ragagnin Oliveira, Nathalie Gaebler Vasconcelos, Simone Simionatto

**Affiliations:** 1 Faculty of Biological and Environmental Sciences, 32279804-970, Federal University of Grande Dourados, Dourados, MS, Brazil; 2 Faculty of Health Sciences, 79800-000, Federal University of Grande Dourados, Dourados, MS, Brazil

## Background

Resistance of *Klebsiella pneumoniae *to carbapenems is mainly associated with acquired carbapenem-hydrolyzing β-lactamases [1]. These β-lactamases can be metallo β-lactamases (IMP, VIM), expanded-spectrum oxacillinases (OXA-48), or Ambler class A enzymes (NMCA, IMI, SME, GES, and KPC) [1,2]. The most common class A carbapenemases in *K. pneumoniae *are the *K. pneumoniae *carbapenemases (KPCs) [2]. Adequate detection of carbapenems resistence genes, such as *bla_KPC_*is crucial for infection control measures and appropriate choice of antimicrobial therapy [3]. In Dourados/MS there are no reports on the monitoring of KPC-producing multiresistant strains of clinical interest. The aim of this study was to identify the gene *bla_KPC _*in strains of *Klebsiella pneumoniae*.

## Methods

The samples were collected during February-May/2012 from patients attended at University hospital, Dourados/MS and identified by classical bacteriological methods. The strains were most frequently obtained from the urinary tract, vaginal secretion, nasal and rectal swab. Antibiograms were realized by using the disk-diffusion method on Mueller-Hinton agar (Bio-Rad Laboratories), and susceptibility break points were determined as previously described and interpreted as recommended by the Clinical and Laboratory Standards Institute [4]. All strains with reduced susceptibility to imipenem or meropenem (MIC, ≥ 2 μg/mL) were screened for carbapenemase production by the modified Hodge test. The presence of gene coding for KPC, were assessed by PCR as described by Cuzon et al., (2010) [5].

## Results and conclusions

From October 2012 to April 2013, 26 strains of *K. pneumoniae carbapenemase *from patients were isolated. Among the wards, those that had a higher incidence of samples were recovered from intensive care units (ICUs) of the hospital, probably due to immune deficiency of patients, submitted to invasive therapeutic procedures. The strains identified as producing carbapenemases were evaluated by PCR amplification using primers specific for *bla_KPC _*gene. Five *K. pneumoniae carbapenemase *strains were positive in PCR. Eighteen strains were positive to modified Hodge test, but were PCR negative. This profile difference could be due to the presence of other classes of carbapenemases. Thus, these eighteen strains of *K. pneumoniae *carbapenemase need to be tested for the presence of β-lactamases such as IMP, VIM, OXA, and NDM-1.
